# Patient priorities in herpes simplex keratitis

**DOI:** 10.1136/bmjophth-2018-000177

**Published:** 2019-04-25

**Authors:** Xiaoxuan Liu, Sai Kolli, Peter McDonnell, Amit Patel, Michael Quinlan, Kevin Skym, Alastair K Denniston, Peter Shah, Geraint P Williams

**Affiliations:** 1 Department of Ophthalmology, University Hospitals Birmingham NHS Foundation Trust, Birmingham, UK; 2 Academic Unit of Ophthalmology, Institute of Inflammation and Ageing, College of Medical and Dental Sciences, University of Birmingham, Birmingham, UK; 3 Birmingham Midlands Eye Centre, Sandwell and West Birmingham Hospitals NHS Trust, Birmingham, UK; 4 Department of Ophthalmology, Heart of England NHS Foundation Trust, Birmingham, UK; 5 Herpes Simplex Keratitis Patient Group, Birmingham, UK; 6 Department of Ophthalmology, Worcestershire Acute Hospitals NHS Trust, Worcester, UK

**Keywords:** herpes simplex keratitis, research priorities, research agenda, patient involvement

## Abstract

**Objective:**

Herpes simplex keratitis (HSK) is a sight-threatening disease and a leading cause of infectious corneal blindness. Involving patients in setting the research agenda maximises patient benefit and minimises research waste. With no published patient involvement exercises, patients’ priorities in HSK are unclear. The objective of this study is to explore patients' priorities for research in HSK.

**Methods:**

A literature review of publications in the year preceding recruitment of patients identified nine domains of research interest. A questionnaire was sent to participants asking them to rank these in order of priority. The ranking results were given a weighted-average score, and a thematic analysis was undertaken for the narrative data.

**Results:**

Thirty-seven patients participated in the survey. Top priorities for patients were risk factors for recurrence of infection, diagnostic tests and treatment failure. The narrative data revealed three key clinical needs: difficulties in long-term symptom control, the need for rapid access care in acute infection and the desire for more accessible information.

**Conclusion:**

This study highlighted three major issues in our current approach to HSK. First, there may be a misalignment between research efforts and patient priorities. Second, high-quality patient information is not widely available. This may hamper patients’ abilities to make informed decisions and contribute towards research. Third, clinical service priorities are of equal importance to patients as research. Researchers and clinicians are encouraged to address both needs in parallel.

**Key messages**

**What is already known about this subject?**

- Involving patients in setting the research agenda ensures research benefits those who ultimately live with a condition and prevents research waste. To date, there are no published patient involvement exercises for herpes simplex keratitis (HSK), and as such it has been difficult to ensure patient priorities are being addressed.

**What are the new findings?**

- This survey would constitute the first published exploration of patients’ priorities for research in HSK. We undertook a patient involvement exercise, conducted in the West Midlands, UK. We found that top research priorities for patients were knowledge of modifiable risk factors for disease recurrence, development of accurate and rapid diagnostic tests, and more understanding of how/when treatment failure occurs.

**How might these results change the focus of research or clinical practice?**

- Our narrative data give a new insight into patients’ urgent clinical needs, which should be addressed in parallel to research. Our group emphasised the need for better symptom control (during and between flare ups), rapid access to specialist ophthalmic care and high-quality patient information resources.

## Introduction

Understanding patients’ perspectives is vital for directing the research agenda. Clinicians, academics and the pharmaceutical industry are all key stakeholders in driving research forward, but their priorities are not always aligned with that of patients.^1^ It has been argued that involving patients in research ensures the benefit to those who ultimately live with the disease and therefore prevents research waste.^2^ In the UK, organisations such as the James Lind Alliance and INVOLVE (a National Institute for Health Research funded advisory group), have been major driving forces in facilitating public involvement in healthcare research.^3^ Similarly, the Patient-Centered Outcomes Research Institute in the the United States was set up to support patient-centred research, and to ensure funding is directed at research questions critical to the patient.^4^

Herpes simplex keratitis (HSK) can be a painful and debilitating disease, and when severe, can take a remitting and relapsing course with gradual loss of sight over time.^5^ The virus is usually acquired early in life, after which it resides in the trigeminal root ganglion in a quiescent state. Years later, the virus travels to the ocular surface via the trigeminal nerve, and has the potential to damage all layers of the cornea.^6^ Making an initial diagnosis of HSK can be difficult due to non-specific clinical signs, as well as low sensitivity and relatively low uptake of corneal PCR assays and conjunctival swabs.^7^ Usually, there is a need to start empirical treatment in the absence of confirmatory tests. Repeated infections can accumulate blinding complications such as scarring, neovascularisation, persistent epithelial defects, corneal melt, neurotrophic keratitis and secondary bacterial infection.^8–10^ The mainstay of treatment is topical antiviral therapy for epithelial disease and/or topical steroids for stromal complications.^11^ Oral antiviral therapy as long-term prophylaxis has been shown to significantly reduce the risk of recurrences, but there is increasing recognition that HSV resistance can occur in up to one-third of patients on oral antiviral therapy for over a year.^12^

There are currently no published studies of the patients’ priorities in research for HSK. The Sight Loss and Vision Priority Setting Partnership produced a list of priorities for research in 12 categories of eye diseases in 2014, however, HSK was not ranked in the list of priorities for corneal and external eye disease.^13^ Patient-reported outcome measures (PROMs) are an assessment of health status that comes directly from the patient and are increasingly used in clinical effectiveness research, and in health policy and commissioning decisions. There is growing interest in the use of PROMs in ophthalmology, however, none have been developed specifically for HSK. Understanding of the patients perspective in ocular surface disease has focused primarily on dry eye disease (DED), and published work is centred on the development of PROMs for symptom control and quality of life (QoL).^14^ Assessment tools, such as the Ocular Surface Disease Index^15^ and Impact of Dry Eye on Everyday Life,^16^ allow assessment of a patient’s QoL and vision-related functioning, and the National Eye Institute Visual Function Questionnaire-25 (VFQ-25) has shown that the degree of visual impairment confers a worse QoL.^17^ While DED and HSK share several QoL implications, there is a wide spectrum of HSK-specific consequences (such as fear of relapse, demanding treatment regimens, neurotrophic keratitis and immunosuppressive treatment) that are not addressed by existing tools.

Reynaud *et al* have published the only QoL study in HSK thus far, focusing on patients during quiescent disease.^18^ They found levels of QoL impairment in quiescent HSK to be comparable with other sight threatening diseases such as anterior uveitis, cataract, graft-versus-host disease and Sjögren-related DED. However, this study was limited by the lack of a QoL tool specific to HSK, and instead used a combination of The National Eye Institute VFQ-25,^19^ the Glaucoma QoL^20^ and the Ocular Surface Disease QoL questionnaire^21^) to assess the various dimensions of living with HSK. From the literature that is currently available, our understanding of the patient’s perspective in HSK is incomplete.

We formed the first HSK patient participation group in the West Midlands, UK with three aims: (1) to recognise patients as stakeholders for research and clinical care in HSK, (2) to provide an opportunity for patients to steer the direction of future research and (3) to understand the patient’s perspective on living with HSK.

## Methods

The survey took place across four regional hospitals in the West Midlands, UK. Patient participation was enrolled by five corneal specialists at clinic appointments. Patients were recruited sequentially during a 6-month time frame. During consultation, all patients with an established diagnosis of HSK were invited to participate in the survey.

A literature search was conducted for publications relating to HSK in the year preceding recruitment of patients (2014). The search strategy was pragmatic in approach and deliberately targeted. Database searches were restricted to PubMed/MEDLINE, with the search term ‘Herpes Simplex Keratitis’ expanded as follows: (‘keratitis, herpetic’[MeSH Terms] OR (‘keratitis’[All Fields] AND ‘herpetic’[All Fields]) OR ‘herpetic keratitis’[All Fields] OR (‘herpes’[All Fields] AND ‘simplex’[All Fields] AND ‘keratitis’[All Fields] OR ‘herpes simplex keratitis’[All Fields]). Date inclusion was: 1 January 2014 to 31 December 2014.

Publications were grouped based on their clinical relevance and classified into nine key domains (online supplementary file). The number of publications for each domain was calculated as a proportion of all research relating to HSK in that year, to serve as an indicator for the research priorities of the scientific community (table 1).

10.1136/bmjophth-2018-000177.supp1 Supplementary data

**Table 1:** Table showing prioritisation of the nine key domains: first from the literature review (52 publications in total) and then from our HSK patient survey

Areas of research interest	Percentage of publications in each area (Number of publications on PubMed for the year 2014)	Areas of research interest by number of publications found on PubMed in 2014 (1=most publications, 7=least publications)	Areas of research interest in order of importance as per HSK survey ranking (1=highest priority, 7=lowest priority)
1. Risk factors for recurrence of infection	**12% (6**)	**4**	**1**
2. How quickly the infection can be treated	**21% (11**)	**2**	**4**
3. When there is failure to treat the infection	**14% (7**)	**3**	**3**
4. Developing tests to guide our treatment more effectively	**8% (4**)	**5**	**2**
5. Uncertainties about disease resistance to treatment	**5% (3**)	**7**	**5**
6. The need for long-term treatment	**7% (4**)	**6**	**8**
7. Risk factors for developing infection	**33% (17**)	**1**	**6**
8. Impact of the disease on quality of life	**0%** (**0**)	**8**	**7**
9. The frequency of hospital visits	**0%** (**0**)	**9**	**9**

All patients in the HSK Patient Participation Group were sent a survey based on these nine domains of research.

For each domain, ranking is given by the number of publications in 2014 as an indicator of where research efforts were focused. This is compared with the ranking of research priorities by our HSK Patient Participation Group survey.

HSK, herpes simplex keratitis.

We distributed the survey online via Survey Monkey (SurveyMonkey, San Mateo, California, USA) for patients who had access to the internet (at https://www.surveymonkey.com; accessed 14 August 2017), and by telephone interview for those who could not access the internet. We asked participants to rank these nine domains of interest in order of importance and share thoughts on each domain. For each patient, the order of the nine domains was randomised automatically by SurveyMonkey. There was also free-text space at the end for patients to comment on anything not covered by the nine domains.

10.1136/bmjophth-2018-000177.supp2 Supplementary data

Ranking results were given a weighted-average score using SurveyMonkey’s standard formula.^22^ Thematic analysis was undertaken by XL and GW for all narrative data using the protocol described by Braun and Clarke.^23^ First, coding of data and identification of potential themes was conducted separately by XL and GW, then key themes were agreed on by consensus. XL, GW and PS defined and named the identified themes and highlighted representative quotes which supported each theme.

## Results

### Priorities of the scientific community

Review of the literature identified nine areas. These nine areas were reworded in plain English for patients: (1) risk factors for recurrence of infection, (2) how quickly the infection can be treated, (3) when there is failure to treat the infection, (4) developing tests to guide our treatment more effectively, (5) uncertainties about disease resistance to treatment, (6) the need for long-term treatment, (7) risk factors for developing infection, (8) impact of the disease on QoL and (9) frequency of hospital visits. The most researched areas were: how quickly the infection can be treated, risk factors for developing infection and when there is failure to treat the infection (table 1).

### Priorities of patients

Fifty-six patients from five centres in the West Midlands agreed to take part in the survey. Participating patients ranged from mild to severe disease, with varying lengths of diagnosis. Forty per cent of participants were male and 60% were female. The mean age of participants was 57 (range 19–89) years. Socioeconomic background was categorised using Index of Multiple Deprivation (IMD) as measured by postal code. Median IMD of the group was 6 (range 1–10).^24^

**Figure 1:**
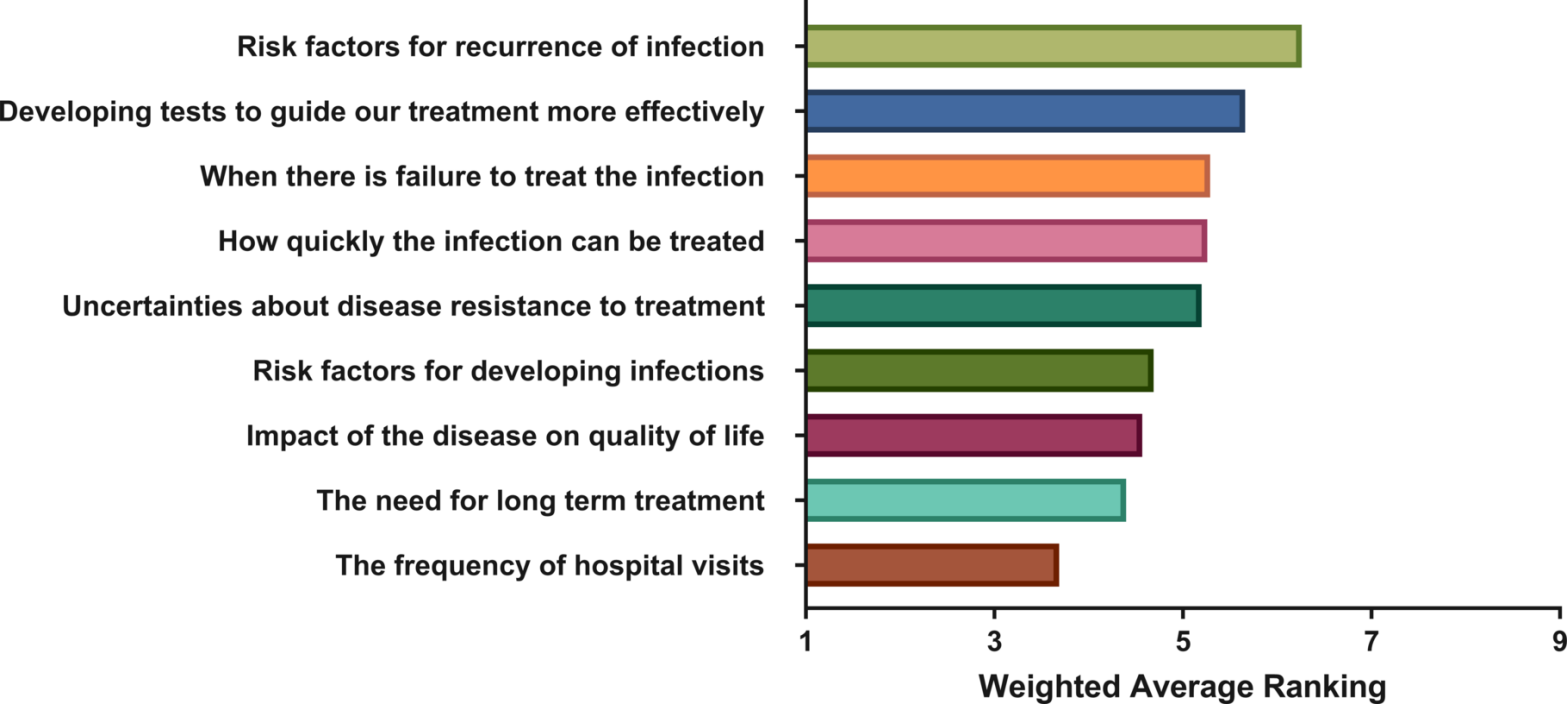
Results of ranking question from HSK patient survey. Weighted-average scores for each domain are shown in order of priority. 9=most important, 1=least important. HSK, herpes simplex keratitis.

Thirty-seven responses to the survey were received, of which six patients completed the questions via telephone. The weighted ranking score of patients for each area of interest is shown in figure 1. The highest rated domains were: risk factors for recurrence of infection (weighted ranking score 6.16), developing tests to guide treatment more effectively (5.57) and when there is failure to treat the infection (5.35). The domain ranked least important was the frequency of hospital visits (3.97).

### Thematic analysis

Using qualitative research techniques and thematic analysis, we also identified three themes from the narrative data.

#### Theme 1: controlling symptoms

A prominent theme was difficulty in controlling symptoms (example quotes in table 2). In many cases, this translated to the need for frequent eye-drops. Acute exacerbations require topical antiviral and steroid therapy every few hours. In patients with complicating DED or high intraocular pressure (IOP), there may be further treatment with lubricating or IOP lowering drops. Some patients describe the practical difficulties with frequent eye drops: ‘I am on fourteen drops a day for the last year, it is difficult for me to keep up,’ and some patients are dependent on others to administer their medication: ‘I rely on my wife to put in my drops because my hands don’t work well—I cannot even use a knife and fork.’ Some symptoms persist despite treatment: ‘The eye is never 80% comfortable even when well. Therefore, long-term treatment would be great.’

**Table 2:** Example quotes from HSK patient survey narrative data

Theme	Example Quotes
**Theme 1: controlling symptoms**	‘I am on 14 drops a day for the last year, it is difficult to keep up’ ‘I rely on my wife to put in my drops because my hands don’t work well’ ‘No matter what treatment you have, the eye is never 80% comfortable’ ‘Sometimes so painful, can’t tolerate light and causes severe headaches’ ‘When I have had the infection in my eye, immediate treatment helps, but my eye is weak for up to a year afterwards’ ‘I have to take drops every day for the last 4 years, it seems unlikely I will ever be free of them’ ‘Worse bit is putting the drops in’ ‘Need to get quicker pain relief’ ‘Anything which would make treatment more effective especially drops, rather than ointment’
**Theme 2:** **access to the ophthalmologist**	‘Early identification for front line non-specialist professionals, for example, Opticians, general practitioners’ ‘Direct access to ophthalmology department without need to involve the general practitioner’ ‘The biggest failure in the system is the inability of general practitioners to recognise it and their misdiagnosis’ ‘Hospital access by request should a worry arise unexpectedly’ ‘Diagnosis needs to be a lot quicker—more specialists need to be assigned’
**Theme 3:** **the need for more information**	**Questions we have answers to: patient education and modifiable risk factors**. ‘How can patients self-identify (recurrence of infection)?’ ‘What are the risk factors and why?’ ‘I’ve never explored or had it explained to me why I got the infection’ ‘Are there any lifestyle factors that could be avoided to prevent recurrence?’ ‘People need to ensure they are not causing or exacerbating risk factors.’ ‘The possibility of resistance to treatment and how to deal with it should be analysed, and the information made available to patients.’ ‘I would like to know how to advise others to prevent them suffering the same infection.’ ‘I think it is also important to support the patient emotionally.’ ‘Is it something you catch or is it something already in your system?’ **Questions we are still looking into: Setting the research agenda** ‘Can it go from one eye to the other?’ ‘If long term treatment will reduce a recurrence happening again?’ ‘(what is the) likelihood of complications, the effectiveness at preventing further recurrences and the necessary duration of treatment?’ ‘What are the risks of long term treatment?’ ‘What can be done to improve outcomes where there has been a late diagnosis and damage has been done?’ ‘Why has (failure to treat the infection) taken place?’ ‘How quickly does catastrophic blindness happen?’ ‘Establishing whether prolonged treatment causes resistance and therefore optimal treatment duration.’

Quotes are grouped into three themes: controlling symptoms, access to the ophthalmologist and the need for more information.

HSK, herpes simplex keratitis.

#### Theme 2: access to the specialist

Participants found it difficult to gain rapid access to the specialist (table 2). In the UK, patients may not have direct access to an ophthalmologist, without referral from a family/general practitioner. One patient commented that ‘the time difference between a general practitioner referral and a consultation appointment is important, and pathology of the disease is not always fully understood at primary care level.’ Others pointed out that ‘diagnosis needs to be a lot quicker—more specialists need to be assigned,’ and that HSK ‘needs to be spotted by normal ophthalmologists.’ It seems that some patients are experiencing significant delays to diagnosis and treatment, which has the potential to cause irreversible damage. One patient suggested developing ‘a treatment pack which can be kept on standby by the patient for instant treatment of flare ups,’ and others asked the question ‘is self-diagnosis acceptable?’

#### Theme 3: the need for more information

In asking the patient for their priorities, we received many questions in return (table 2). We have categorised these into two groups: questions we have the answer to (which should be made widely available as patient information) and questions without clear answers (which should form the basis of setting the research agenda).

Questions ranged from ‘can it go from one eye to the other?’ and ‘what the risk factors are and why?’ to more challenging ones such as ‘why has (failure to treat the infection) taken place?’ and ‘what can be done to improve outcomes when there has been a late diagnosis and damage has been done?’ Some patients pointed out the need for information early on: ‘I’ve never had it explained to me why I got the infection’ and ‘how quickly does catastrophic blindness happen?’ Many questions also centred around lifestyle changes and modifiable risk factors: ‘Are there any lifestyle factors that could be avoided to prevent recurrence?’ as well as ways in which patients can play a more active role in managing their disease: ‘How can patients self-identify (recurrence of infection)?’

## Discussion

With a large clinical and economic impact, significant research efforts are directed towards HSK—including the development of better diagnostic tools, treatment strategies and vaccination.^25–28^ To our knowledge, this study is the first published report of patient priorities for HSK research. Through this qualitative exercise, we have begun to identify what is most important for the patient. Our literature search highlighted disparities between the research priorities of patients and the scientific community.

The top priorities for research were: risk factors for recurrence of infection; developing tests to guide treatment more effectively and failure to treat the infection. Significant efforts have already been made to understand the viral and host factors influencing infection and reactivation, but this remains poorly understood. We know that certain triggers such as hormonal changes, fever, psychological stress and ultraviolet light exposure may induce reactivation, but the underlying mechanisms remain unclear. Improving diagnosis and monitoring with the use of novel imaging techniques has been an area of rapid growth in recent years and continues to expand. Newer techniques such as anterior segment optical coherence tomography and in vivo confocal microscopy, as well as automated imaging analysis platforms are providing more accurate ways of visualising and quantifying disease.^28–30^ Treatment failure results in irreversible scarring which may require corneal grafting to preserve vision. The complicating factor in HSK is that the insult of surgery itself may trigger viral reactivation and cause graft failure. Research efforts have focused on understanding the mechanisms of graft rejection and effectiveness of antiviral treatment to reduce the risk of graft failure.^31^ Surprisingly, frequency of hospital visits was ranked lowest in priority. One patient commented that ‘regular and predictable consultations not only provide reassurance, but a better chance of correct treatment,’ while another asked ‘would less time between visits help catch it earlier?’ It is worth considering whether more routine reviews improve outcomes, or if it is a source of reassurance for patients. This is especially important, as anxiety impairs QoL in patients with HSK even while in remission.^18^

An unintended outcome of this study was the extent to which patients also used the survey to draw attention to their priorities for clinical care. They highlighted significant areas of unmet need such as poor access to specialists during times of acute infection. Patients asked if it was acceptable to ‘self-diagnose’ acute infection and use ‘rescue treatment packs’ at home. In chronic obstructive pulmonary disease, National Institute for Health and Care Excellence guidelines recommends the use of rescue packs containing steroids and antibiotics kept at home for acute exacerbations.^32^ A similar set-up in HSK may benefit patients in acute infection, however, we need strategies that enable them to do so safely.

An area requiring attention is patient education. High-quality information provision is an intervention which has been shown to positively impact patients’ experiences and health behaviours. As such, providing accessible information is now firmly embedded in health policy.^33^ Currently, there is little in the way of published literature and digital resources for HSK. Efforts should concentrate on patient education that is effective, engaging and accessible for the wider population.

This study represents the first open invitation for patients with HSK to express what is most important to them, as well as the first examination of whether current research is aligned with the priorities of patients. Despite being a leading cause of infectious corneal blindness, HSK did not feature in the top priorities ranked in the corneal and external diseases category of the Sight Loss and Vision Priority Setting Partnership Survey. From the disparity of research priorities demonstrated by our study, we feel that a more in-depth priority setting exercise for HSK patients would be beneficial in directing future research.

Our study has several limitations. Our initial scope of the literature which informed the 9 domains of research interest only included studies in the year preceding our survey. This was not intended to be an exhaustive review of the literature, but rather an indication of where research efforts within HSK were focused at the time. Nineteen patients who originally agreed to take part did not complete the survey and it is unclear whether the drop-out rate may have introduced bias to our findings. For this initial qualitative scoping exercise, we have not collected baseline characteristics of participants, therefore, further evaluation is required to determine the needs and priorities for different patient groups. Our study suggests that elderly patients have concerns specific to coping with the demands of frequent drops. With an ageing population, it is vital that clinicians consider the elderly patient’s physical and cognitive limitations, and the feasibility of their management plans.^34^ Furthermore, centre and patient variation in treatment regimen may exist, including, for example, the need to treat concurrent elevation in IOP, which may have exacerbated the need for drop frequency. More in-depth interviews among different patient groups are required to explore the full spectrum of patient priorities. Our geographical reach was limited to the West Midlands, UK, where guidelines and structure of service provision differ from other areas. It is, however, an area of ethnic and socioeconomic diversity, with the largest non-white regional population outside of London.^35^ Further investigation encompassing the wider cohort is needed.

This study has highlighted three issues in our current approach to HSK. First, the research agenda has so far been set without published knowledge on what is important for the patient. This can lead to a disparity between priorities of patients and the scientific community. We have identified several areas of key importance to patients for future research to bridge this gap. Second, there is a lack of easily accessible patient information on HSK. This hampers their ability to make informed decisions relating to their own clinical care and limits their ability to contribute towards research. Third, clinical service priorities are of equal importance to patients as research. Clinicians and researchers should be aware that a patient’s urgent desire for better clinical care today may outweigh the uncertain benefits of research tomorrow, even if the latter might lead to a cure. To understand patients’ true priorities, the enquiry must be open and unbiased.

Our survey is the first attempt to engage patients with HSK in a dialogue about setting research priorities. Patients are eager to fulfil their role as key stakeholders for research, therefore, it is vital that patients are provided with a platform to voice their priorities and establish partnerships with researchers. We intend to expand our patient participation group for this purpose and continue to share our findings with the scientific and clinical community.
