# Supplementary material for: Patient priorities in herpes simplex keratitis
Source: BMJ Open Ophthalmol. 2019 Apr 25;4(1):e000177. doi: 10.1136/bmjophth-2018-000177 (PMC6528774; doi:10.1136/bmjophth-2018-000177)
Supplement: Supplementary data [file bmjophth-2018-000177supp001.docx]

**Supplementary Table.**

Results of PubMED/MEDLINE literature review of research publications relating to HSK in 2014, grouped into 9 key domains.

| **HSK Summary PubMed 2014** | **Research Priority** |
| --- | --- |
| Trufanov SV, Malozhen SA, Zaĭtsev AV. [Lamellar minikeratoplasty for recurrent herpes keratitis in the late post-LASIK period]. Vestn Oftalmol. 2014 Sep-Oct;130(5):60-3.  PMID: 25711064. | 3. When there is failure to treat the infection |
| Sun YZ, Guo L, Zhang FS. Curative effect assessment of bandage contact lens in neurogenic keratitis. Int J Ophthalmol. 2014 Dec 18;7(6):980-3.  PMID: 25540750; | 6. The need for long term treatment |
| Alekseev O, Limonnik V, Donovan K, et al. Activation of  checkpoint kinase 2 is critical for herpes simplex virus type 1 replication in corneal epithelium. Ophthalmic Res. 2015;53(2):55-64.  PMID: 25531207 | 7. Risk factors for developing infections |
| Toriyama K, Inoue T, Suzuki T, et al. Necrotizing keratitis caused by acyclovir-resistant herpes simplex virus. Case Rep Ophthalmol. 2014 Oct  10;5(3):325-8.  PMID: 25473399 | 5. Uncertainties about disease resistance to treatment |
| Park PJ, Chang M, Garg N, et al. Corneal lymphangiogenesis  in herpetic stromal keratitis. Surv Ophthalmol. 2015 Jan-Feb;60(1):60-71.  PMID: 25444520 | 2. How quickly the infection can be treated |
| Buela KA, Hendricks RL. Cornea-infiltrating and lymph node dendritic cells contribute to CD4+ T cell expansion after herpes simplex virus-1 ocular infection. J Immunol. 2015 Jan 1;194(1):379-87.  PMID: 25422507 | 1. Risk Factors for recurrence of infection |
| Gaddipati S, Estrada K, Rao P, et al. IL-2/anti-IL-2 antibody complex treatment inhibits the development but not the progression of herpetic stromal keratitis. J Immunol. 2015 Jan 1;194(1):273-82.  PMID: 25411200 | 7. Risk factors for developing infections |
| Kanellopoulos AJ, Asimellis G. Clinical Correlation between Placido, Scheimpflug and LED Color Reflection Topographies in Imaging of a Scarred Cornea. Case Rep Ophthalmol. 2014 Oct 1;5(3):311-7.  PMID: 25408671 | 4. Developing tests to guide our treatment more effectively |
| Li Z, Duan F, Lin L, et al. A new approach of delivering siRNA to the cornea and its application for inhibiting herpes simplex keratitis. Curr Mol Med. 2014;14(9):1215-25.  PubMed PMID: 25336327. | 2. How quickly the infection can be treated |
| Seo Y, Kim MK, Lee JH, et al. Expression of Lymphangiogenic Markers in Rejected Human Corneal Buttons after Penetrating Keratoplasty. Curr Eye Res. 2015 Sep;40(9):902-12.  PMID: 25330436. | 3. When there is failure to treat the infection |
| Cottrell P, Ahmed S, James C, et al. Neuron J is a rapid and reliable open source tool for evaluating corneal nerve density in herpes simplex keratitis. Invest Ophthalmol Vis Sci. 2014 Oct 16;55(11):7312-20  PMID: 25324286. | 4. Developing tests to guide our treatment more effectively |
| Arora T, Sharma N, Arora S, et al. Fulminant herpetic keratouveitis with flap necrosis following laser in situ keratomileusis: Case report and review of literature. J Cataract Refract Surg. 2014 Dec;40(12):2152-6  PMID: 25311411. | 1. Risk Factors for recurrence of infection |
| Stanzel TP, Diaz JD, Mather R, et al. The epidemiology of herpes simplex virus eye disease in Northern California. Ophthalmic Epidemiol. 2014 Dec;21(6):370-7  PMID: 25299934. | 7. Risk factors for developing infections |
| Bryant-Hudson KM, Gurung HR, Zheng M, et al. Tumor necrosis factor alpha and interleukin-6 facilitate corneal lymphangiogenesis in response to herpes simplex virus 1 infection. J Virol. 2014 Dec;88(24):14451-7.  PMID: 25297992 | 2. How quickly the infection can be treated |
| Rolinski J, Hus I. Immunological aspects of acute and recurrent herpes simplex keratitis. J Immunol Res. 2014.  PMID: 25276842 | 1. Risk Factors for recurrence of infection |
| Molesworth-Kenyon SJ, Milam A, Rockette A, et al. Expression, Inducers and Cellular Sources of the Chemokine MIG (CXCL 9), During Primary Herpes Simplex Virus Type-1 Infection of the Cornea. Curr Eye Res. 2015;40(8):800-8.  PMID: 25207638. | 7. Risk factors for developing infections |
| Chou TY, Hong BY. Ganciclovir ophthalmic gel 0.15% for the treatment of acute herpetic keratitis: background, effectiveness, tolerability, safety, and future applications. Ther Clin Risk Manag. 2014 Aug 20;10:665-81  PMID: 25187721 | 2. How quickly the infection can be treated |
| Petrera E, Níttolo AG, Alché LE. Antiviral action of synthetic stigmasterol derivatives on herpes simplex virus replication in nervous cells in vitro. Biomed Res Int. 2014.  PMID: 25147828 | 2. How quickly the infection can be treated |
| Larrañaga Fragoso P, Boto de Los Bueis A, Bravo Ljubetic L, et al. Herpes Simplex Keratitis in Rheumatoid Arthritis Patients. Ocul Immunol Inflamm. 2016 Jun;24(3):282-7.  PMID: 25140583. | 1. Risk Factors for recurrence of infection |
| Domínguez-López A, Bautista-de Lucio VM, Serafín-López J, et al. Amniotic membrane modulates innate immune response inhibiting PRRs expression and NF-κB nuclear translocation on limbal myofibroblasts. Exp Eye Res.  2014 Oct;127:215-23.  PMID: 25117451. | 3. When there is failure to treat the infection |
| Petrovski G, Pasztor K, Orosz L, et al. Herpes simplex virus types 1 and 2 modulate autophagy in SIRC corneal cells. J Biosci. 2014 Sep;39(4):683-92.  PMID: 25116622. | 7. Risk factors for developing infections |
| Paavilainen H, Romanovskaya A, Nygårdas M, et al. Innate responses to small interfering RNA pools inhibiting herpes simplex virus infection in astrocytoid and epithelial cells. Innate Immun. 2015 May;21(4):349-57.  PMID: 24996409. | 2. How quickly the infection can be treated |
| Sharma S, Rajasagi NK, Veiga-Parga T, et al. Herpes virus entry mediator (HVEM) modulates proliferation and activation of regulatory T cells following HSV-1 infection. Microbes Infect. 2014 Aug;16(8):648-60.  PMID: 24956596. | 2. How quickly the infection can be treated |
| Shaibu AM, Aminu M, Musa BO, et al. Seroprevalence of IgG antibodies to herpes simplex virus type-1 in Nigerian children. Niger J Med. 2014 Jan-Mar;23(1):40-5.  PMID: 24946453. | 7. Risk factors for developing infections |
| Brown CR, Wagoner MD, Welder JD, et al. Boston keratoprosthesis type 1 for herpes simplex and herpes zoster keratopathy. Cornea. 2014 Aug;33(8):801-5.  PMID: 24932767. | 3. When there is failure to treat the infection |
| Reddy PB, Sehrawat S, Suryawanshi A, et al. An approach to control relapse of inflammatory lesions after discontinuation of primary therapy. PLoS One. 2014 May 20;9(5):e98051.  PMID: 2484612. | 6. The need for long term treatment |
| Matundan H, Mott KR, Ghiasi H. Role of CD8+ T cells and lymphoid dendritic cells in protection from ocular herpes simplex virus 1 challenge in immunized mice. J Virol. 2014 Jul;88(14):8016-27.  PMID: 24807710 | 1. Risk Factors for recurrence of infection |
| Groupe bibliographique de la SPILF. [Acyclovir resistance of HSV in immunocompetent patients: is prophylaxis incriminated?]. Med Mal Infect. 2014 Mar;44(3):134-5.  PMID: 24804311. | 5. Uncertainties about disease resistance to treatment |
| Yun H, Rowe AM, Lathrop KL, et al. Reversible nerve damage and corneal pathology in murine herpes simplex stromal keratitis. J Virol. 2014 Jul;88(14):7870-80.  PMID: 24789786 | 2. How quickly the infection can be treated |
| Allen SJ, Mott KR, Ghiasi H. Inhibitors of signal peptide peptidase (SPP) affect HSV-1 infectivity in vitro and in vivo. Exp Eye Res. 2014 Jun;123:8-15.  PMID: 24768597 | 2. How quickly the infection can be treated |
| Alekseev O, Donovan K, Limonnik V, et al. Nonthermal Dielectric Barrier Discharge (DBD) Plasma Suppresses Herpes Simplex Virus Type 1 (HSV-1) Replication in Corneal Epithelium. Transl Vis Sci Technol. 2014 Mar 27;3(2):2.  PMID: 24757592 | 2. How quickly the infection can be treated |
| Kim IJ, Saied AA, Chouljenko VN, et al. Functional  hierarchy of herpes simplex virus type-1 membrane proteins in corneal infection and virus transmission to ganglionic neurons. Curr Eye Res. 2014 Dec;39(12):1169-77  PMID: 24749493. | 7. Risk factors for developing infections |
| Sacchetti M, Lambiase A. Diagnosis and management of neurotrophic keratitis. Clin Ophthalmol. 2014 Mar 19;8:571-9  PMID: 24672223 | 3. When there is failure to treat the infection |
| Sharma A, Mohan K, Sharma R, et al. Alopecia following oral acyclovir for the treatment of herpes simplex keratitis. Middle East Afr J Ophthalmol. 2014 Jan-Mar;21(1):95-7  PMID: 24669156 | 6. The need for long term treatment |
| Allen SJ, Mott KR, Ghiasi H. Overexpression of herpes simplex virus glycoprotein K (gK) alters expression of HSV receptors in ocularly-infected mice. Invest Ophthalmol Vis Sci. 2014 Apr 15;55(4):2442-51  PMID: 24667863 | 7. Risk factors for developing infections |
| Thompson RL, Williams RW, Kotb M, et al. A forward phenotypically driven unbiased genetic analysis of host genes that moderate herpes simplex virus virulence and stromal keratitis in mice. PLoS One. 2014 Mar 20;9(3):e92342.  PMID: 24651695 | 7. Risk factors for developing infections |
| Huang J, Kadonosono K, Uchio E. Antiadenoviral effects of ganciclovir in types inducing keratoconjunctivitis by quantitative polymerase chain reaction methods. Clin Ophthalmol. 2014 Jan 30;8:315-20.  PMID: 24511226 | 4. Developing tests to guide our treatment more effectively |
| Knickelbein JE, Buela KA, Hendricks RL. Antigen-presenting cells are stratified within normal human corneas and are rapidly mobilized during ex vivo viral infection. Invest Ophthalmol Vis Sci. 2014 Feb 24;55(2):1118-23.  PMID: 24508792 | 7. Risk factors for developing infections |
| Kinchington PR. Targeting host pathways to block HSV-1 at the cornea. Invest Ophthalmol Vis Sci. 2014 Feb 3;55(2):716. doi: 10.1167/iovs.14-13879.  PMID: 24492207. | 2. How quickly the infection can be treated |
| Trikha S, Parikh S, Osmond C, et al. Long-term outcomes of Fine Needle Diathermy for established corneal neovascularisation. Br J Ophthalmol. 2014 Apr;98(4):454-8.  PMID: 24457357. | 3. When there is failure to treat the infection |
| Tappeiner C, Heiligenhaus A. [Keratitis caused by herpes simplex and varicella zoster virus]. Klin Monbl Augenheilkd. 2014 Jan;231(1):79-93;  PMID: 24443138 | 7. Risk factors for developing infections |
| West DM, Del Rosso CR, Yin XT, et al. CXCL1 but not IL-6 is required for recurrent herpetic stromal keratitis. J Immunol. 2014 Feb 15;192(4):1762-7.  PMID: 24442436. | 7. Risk factors for developing infections |
| Li J, Ma H, Zhao Z, et al. Deep anterior lamellar keratoplasty using precut anterior lamellar cap for herpes simplex keratitis: a  long-term follow-up study. Br J Ophthalmol. 2014 Apr;98(4):448-53.  PMID: 24420918. | 3. When there is failure to treat the infection |
| Saied AA, Chouljenko VN, Subramanian R, et al. A replication competent HSV-1(McKrae) with a mutation in the amino-terminus of glycoprotein K (gK) is unable to infect mouse trigeminal ganglia after cornea infection. Curr Eye Res. 2014 Jun;39(6):596-603.  PMID: 24401006. | 7. Risk factors for developing infections |
| Borkar DS, Gonzales JA, Tham VM, et al. Association between atopy and herpetic eye disease: results from the  pacific ocular inflammation study. JAMA Ophthalmol. 2014 Mar;132(3):326-31  PMID: 24370901. | 7. Risk factors for developing infections |
| Alekseev O, Donovan K, Azizkhan-Clifford J. Inhibition of ataxia telangiectasia mutated (ATM) kinase suppresses herpes simplex virus type 1 (HSV-1) keratitis. Invest Ophthalmol Vis Sci. 2014 Feb 3;55(2):706-15.  PMID: 24370835 | 7. Risk factors for developing infections |
| Cao T, Xing Y, Yang Y, et al. Correlation between matrix metalloproteinase expression and activation of the focal adhesion kinase signaling pathway in herpes stromal keratitis. Exp Ther Med. 2014 Jan;7(1):280-286. Epub 2013 Nov 13.  PMID: 24348806. | 7. Risk factors for developing infections |
| Xu K, Liu XN, Zhang HB, et al. Replication-defective HSV-1 effectively targets trigeminal ganglion and inhibits viral pathopoiesis by mediating interferon gamma expression in SH-SY5Y cells. J Mol Neurosci. 2014 May;53(1):78-86.  PMID: 24347277. | 1. Risk Factors for recurrence of infection |
| Vadlapudi AD, Cholkar K, Vadlapatla RK, et al. Aqueous nanomicellar formulation for topical delivery of biotinylated lipid prodrug of acyclovir: formulation development and ocular biocompatibility. J Ocul Pharmacol Ther. 2014 Feb;30(1):49-58.  PMID: 24192229 | 6. The need for long term treatment |
| Barrado L, Suarez MJ, Pérez-Blázquez E, et al. Could polymerase chain reaction tests on conjunctival swabs be useful to diagnose herpetic keratitis? Enferm Infecc Microbiol Clin. 2014 Jan;32(1):28-30.  PMID: 24060450. | 4. Developing tests to guide our treatment more effectively |
| Pan D, Kaye SB, Hopkins M, et al. Common and new  acyclovir resistant herpes simplex virus-1 mutants causing bilateral recurrent herpetic keratitis in an immunocompetent patient. J Infect Dis. 2014 Feb 1;209(3):345-9.  PMID: 23945375 | 5. Uncertainties about disease resistance to treatment |
| Zhao G, Chen H, Song Z, et al. Glial fibrillary acidic protein  expression during HSV-1 infection in mouse cornea. APMIS. 2014 Feb;122(2):128-35.  PMID: 23758602. | 7. Risk factors for developing infections |
| **Total** | **52** |
