# Supplementary material for: Patient priorities in herpes simplex keratitis
Source: BMJ Open Ophthalmol. 2019 Apr 25;4(1):e000177. doi: 10.1136/bmjophth-2018-000177 (PMC6528774; doi:10.1136/bmjophth-2018-000177)
Supplement: Supplementary data [file bmjophth-2018-000177supp002.pdf]

Welcome to the West Midlands Herpes Simplex Keratitis Patient Involvement Group

Thank you for agreeing to take part in this questionnaire.

* 1. Please rank the following areas of research in Herpes Simplex Keratitis by order of importance (1-9). 9 being the most important, and 1 being the least important.

- [ ] How quickly the infection can be treated.
- [ ] The frequency of hospital visits.
- [ ] The need for long term treatment.
- [ ] When there is failure to treat the infection.
- [ ] Uncertainties about disease resistance to treatment.
- [ ] Risk factors for developing infection.
- [ ] Risk factors for recurrence of infection.
- [ ] Impact of the disease on quality of life.
- [ ] Developing tests to guide our treatment more effectively.

# For each of the following topics, please explain any specific points you would like us to research.

* 2. Regarding each topic area, please explain any specific points you would like us to research.

How quickly the infection can be treated.

The frequency of hospital visits.

The need for long term treatment.

When there is failure to treat the infection.

Uncertainties regarding disease resistance to treatment.

Risk factors for developing the infection.

Risk factors for recurrence of infection.

Impact of the disease on quality of life.

Developing tests to guide treatment more effectively.

Other:
